# How hard is it to tell which is a Condorcet committee?^☆^

**DOI:** 10.1016/j.mathsocsci.2013.06.004

**Published:** 2013-11

**Authors:** Andreas Darmann

**Affiliations:** University of Graz, Institute of Public Economics, Universitaetsstr. 15, 8010 Graz, Austria

## Abstract

This paper establishes the computational complexity status for a problem of deciding on the quality of a committee. Starting with individual preferences over alternatives, we analyse when it can be determined efficiently if a given committee C satisfies a weak (resp. strong) Condorcet criterion–i.e., if C is at least as good as (resp. better than) every other committee in a pairwise majority comparison. Scoring functions used in classic voting rules are adapted for these comparisons. In particular, we draw the sharp separation line between computationally tractable and intractable instances with respect to different voting rules. Finally, we show that deciding if there exists a committee which satisfies the weak (resp. strong) Condorcet criterion is computationally hard.

## Highlights

• We focus on the complexity to decide if a committee satisfies a Condorcet criterion.
• For Borda voting this decision problem is coNP-complete.
• For approval voting, we draw the separation line between hard and easy instances.
• It is coNP-hard to decide if there is a weak (resp. strong) Condorcet committee.

## 1 Introduction

### 1.1 Introduction

There are several approaches to determine a single winner of an election, given the preferences of a set of voters over a set of alternatives. Centuries ago, Condorcet (1785) suggested the winner to be the alternative that is preferred to every other alternative by a majority of voters if such an alternative exists. A winner of these pairwise majority comparisons is called Condorcet winner. Voting procedures that elect such a candidate (if one exists) and the set of Condorcet winners in general, have been extensively studied (see, e.g.,  Brams and Fishburn, 2002; Fishburn, 1977; Nurmi, 1987).

A natural generalization of a Condorcet winner to committees, i.e., sets of alternatives of a given size, is proposed by Fishburn (1981). There, it is assumed that the voters have preferences over committees that satisfy certain conditions. In particular, Fishburn (1981) defines a *Condorcet committee* as a committee that is preferred to every other committee by a majority of voters. In contrast, Gehrlein (1985) considers the single members of the committee instead of the entire group. In the terminology of Gehrlein (1985), a committee is a Condorcet committee if each element in the set beats each element not in the set in terms of a pairwise majority comparison. Kaymak and Sanver (2003) link these two approaches by means of certain “extension-axioms”. Another approach, with a certain vicinity to both Fishburn’s and Gehrlein’s approach, is proposed by Elkind et al. (2011). Like Gehrlein, they start with rankings of the alternatives; on this basis, a subset of the alternatives is defined to be a *Condorcet winning set* if for each element x not in the set, a majority of voters prefers at least one of the set’s elements to x. In particular, Elkind et al. (2011) investigate the minimum size of Condorcet winning sets.

Other approaches, however, are taken by Ratliff (2006) and Brams et al. (2006). Ratliff (2006) argues that the Dodgson voting rule and the Kemeny voting rule should be generalized to compare sets of alternatives. Brams et al. (2006) use the Hamming distance to find a committee that is “closest” to the voters’ favourite committee, where closeness is evaluated by means of a minimax and minisum criterion.

In recent papers, the problem of selecting committees is extended by additionally taking into account weight constraints; see, e.g., Delort et al. (2011) and Klamler et al. (2012).

Similarly to Gehrlein (1985) and Elkind et al. (2011), we start with individual preferences over alternatives. It is assumed that the voters’ preferences are additively separable, i.e., the value a committee bears for a voter is determined by the sum of the candidates’ values (scores) for the voter. Here, the score of a voter is evaluated by means of scoring functions adapted from classic voting rules such as approval voting and Borda voting. Like Fishburn (1981), however, we focus on a Condorcet k-committee in the weak and strong sense respectively — i.e., a committee of a given size k that is at least as good as (resp. better than) each other committee of the same size in a pairwise majority comparison.

### 1.2 Related work and contribution of this paper

In line with, e.g., Bartholdi et al. (1989), Procaccia et al. (2008), and Betzler et al. (2011), we focus on the computational complexity in connection with voting schemes. As also Procaccia et al. (2008) point out, voting schemes (be it to elect a single winner, be it to elect a committee) may–despite showing some desirable properties–have the severe drawback that it may be computationally hard to tell who finally won the election. Thus, the practical applicability of such schemes is strongly limited. For this reason, it is useful to classify voting schemes into complexity classes: loosely speaking, into the class of problems for which polynomial algorithms are known (*computationally tractable* problems), and into the class of problems for which polynomial algorithms seem unlikely to exist (NP-hard or coNP-hard problems; these problems are called *computationally intractable*).

In the literature, multi-winner (or committee) selection problems in proportional representation voting schemes have been considered from a computational point of view, the main attention drawn to the schemes of Monroe and Chamberlin–Courant. Typically, the misrepresentation of a voter is evaluated by means of numerical scores used in positional voting rules, with particular focus on approval and Borda scores (see, e.g.,  Brams and Potthoff, 1998; Boutilier and Lu, 2011; Procaccia et al., 2008; Betzler et al., 2011). In Procaccia et al. (2008) and Boutilier and Lu (2011), hardness results for the multi-winner selection problem are obtained, when the goal is to minimize total misrepresentation. Procaccia et al. (2008) prove that in both the Monroe and Chamberlin–Courant schemes, the problem is NP-complete even for the approval misrepresentation function, i.e., if approvals are used as misrepresentation values. Boutilier and Lu (2011) show that for the Chamberlin–Courant scheme, it is NP-complete, if the misrepresentation values are given by Borda scores. In addition to minimizing total misrepresentation, Betzler et al. (2011) investigate also the multi-winner selection problem when the goal is to minimize the maximum misrepresentation. Betzler et al. (2011) show that these minimax versions of the two schemes are NP-complete as well, for both the approval and the Borda misrepresentation function. Besides, Betzler et al. (2011) show that in case of single-peaked preferences and arbitrary misrepresentation functions, the complexity status of the problem turns from NP-complete to polynomial-time solvable in the Chamberlin–Courant scheme, for both minimum total misrepresentation and minimax misrepresentation, and in the Monroe scheme for minimax misrepresentation.

Besides, Procaccia et al. (2007) consider the problem of finding a coalition (committee) with the maximum number of supporters under the use of yes–no voting, originally introduced by Brams and Fishburn (1993). Even if each member (voter) i is asked only to name a set $N_{i}$ of parties (candidates) she wishes to be excluded from a coalition (and thus supports a coalition C iff $C\cap N_{i}=0̸$), it is shown that it is NP-hard to find a coalition with the maximum number of supporters under the restriction that the size of the coalition exceeds a given lower bound. It should be noted that such a winning coalition of size k, even if it is unique, is not necessarily a winner in terms of the Condorcet criteria used in our paper, and vice versa.

However, in their seminal paper, Bartholdi et al. (1989), investigate the computational complexity involved in deciding if a given candidate is a single winner of an election. In particular, they show that for both Dodgson rule and Kemeny rule this decision problem is NP-hard. We follow this approach and investigate the computational complexity involved in deciding whether a given committee is a weak respectively strong Condorcet k-committee.

As mentioned above, our evaluation process is mainly based on the usage of approval and Borda scores, which are used in many of the related papers, e.g., in Brams and Potthoff (1998), Boutilier and Lu (2011), Procaccia et al. (2008), and Betzler et al. (2011). In this paper, with our main results we show that deciding if a given committee is a weak (resp. strong) Condorcet k-committee is coNP-complete for Borda voting, and draw the sharp separation line between computationally tractable and intractable cases with respect to approval voting. Therewith, we establish the computational complexity status for these problems for the most reasonable voting processes. In a different framework, namely in the context of maximin fairness in connection with spanning trees, results of the same flavour are given by Darmann et al. (2009, 2010). Finally, we show that the related natural decision problem if a strong Condorcet k-committee exists is coNP-hard, and that deciding if there is a weak Condorcet k-committee is both NP-hard and coNP-hard.

### 1.3 Organization of the paper

This work is structured as follows. In Section  2 we introduce the formal framework including scoring functions and problem definitions. In Section  3 we state our computational complexity results for the decision problems if a given k-committee is a weak (resp. strong) Condorcet k-committee: Section  3.1 deals with Borda voting, in Section  3.2 we analyse the computational complexity with respect to approval voting, and we consider the case of multichotomous preferences in Section  3.3. Section  4 deals with the related questions whether a weak respectively strong Condorcet k-committee exists. The paper ends with some concluding remarks in Section  5.

## 2 Preliminaries

### 2.1 Binary relations and preference classes

Throughout this work, a binary relation ≿ on a set B is called *complete* if ∀a,b∈B,a≠b, a≿b or b≿a. It is called *reflexive* if ∀b∈B,b≿b. We call ≿ *transitive* if ∀a,b,c∈B,(a≿b and b≿c)⇒a≿c. Relation ≿ is called *asymmetric* if ∀a,b∈B,a≿b⇒¬(b≿a). Finally, ≿ is *symmetric* if ∀a,b∈B, a≿b⇒b≿a.

In the course of this paper, we will use the terms preference relation and binary relation interchangeably. A binary relation is called *weak order* if it is complete, reflexive and transitive. A *strict order* is a complete, transitive and asymmetric binary relation.

In what follows, let I={1,2,…,r} denote a finite set of voters and A be a finite set of alternatives (or candidates). It is assumed that every voter i,1≤i≤r, states her preferences over the alternatives in terms of a complete and transitive preference relation ${≿}_{i}$ on A. Note that   ${≿}_{i}$ consists of an asymmetric part ${≻}_{i}$ and a symmetric part ${∼}_{i}$ respectively.

The symmetric part ${∼}_{i}$ of ${≿}_{i}$ induces a partition $A_{1},A_{2},\ldots ,A_{q}$ of A, such that for all j,1≤j≤q, we have $a{∼}_{i}b$ for all $a,b\in A_{j}$. The sets $A_{j}$, 1≤j≤q, are called preference classes. If q≥2 the order ${≿}_{i}$ is called multichotomous; we call ${≿}_{i}$ dichotomous in the case q=2, and trichotomous in the case q=3. Furthermore, we refer to the r-tuple $\pi =\left({≿}_{1},{≿}_{2},\ldots ,{≿}_{r}\right)$ as a voter preference profile.

### 2.2 Scoring functions and committees

In our framework, voting procedures are used to evaluate the individual preference relations. In particular, we make use of scoring functions, which can be seen as a generalization of the positional scoring procedures (for details concerning these procedures see  Brams and Fishburn, 2002).

Definition 2.1 For i∈I, a function $v_{i}:A\to N_{0}$ is called voter i’s scoring function, if for all $a,b\in A\;a{≿}_{i}b\Leftrightarrow v_{i}\left(a\right)\geq v_{i}\left(b\right)$.

In a very simple setting, voters are allowed to distinguish between “good” and “bad” candidates only; i.e., each i∈I, partitions the candidate set A into a set $S_{i}\subset A$ of edges individual i approves of and a set $S_{i}^{c}≔A\setminus S_{i}$ individual i disapproves of (see  Brams and Fishburn, 1983).

Definition 2.2 In approval voting, for each i∈I voter i’s scoring function is the function $v_{i}:A\to N_{0}$ with

$$v_{i}\left(a\right)=\left\{ \begin{array}{cc} 1 & \text{if }a\in S_{i}\\ 0 & \text{if }a\in S_{i}^{c}\text{.} \end{array}\right.$$

Approval voting under the requirement that, for a given $t\in N,\left|S_{i}^{c}\right|=t$ for 1≤i≤r is called t*-veto*. Given t∈N, t*-approval*corresponds to approval voting in the case $\left|S_{i}\right|=t$ for all 1≤i≤r. 1-approval is called *plurality voting*, 1-veto is denoted by *antiplurality voting* (for details we refer the reader to  Brams and Fishburn, 2002; Roberts, 1991).

Another well-studied voting procedure is Borda voting, a central representative of the positional scoring procedures used in voting theory (see  Brams and Fishburn, 2002). For Borda voting we assume that the individual preferences are stated in terms of strict^1^orders over A; in our terminology the numerical evaluation used by this procedure is captured as follows.

Definition 2.3 Let π consist of strict orders over A. In Borda voting, for each i∈I voter i’s scoring function is the function $v_{i}:A\to N_{0}$ defined by $v_{i}\left(a\right)=\left|\left\{b\in A\setminus \left\{a\right\}:\;a{≻}_{i}b\right\}\right|$.

Given a∈A, in approval voting (Borda voting) we refer to the value $v_{i}\left(a\right)$ as voter i’s *approval count (Borda count)* of a.

Given k∈N, let $A_{k}≔\left\{C\subseteq A:\left|C\right|=k\right\}$ be the set of k-element subsets of A. Such a subset (i.e., an element of $A_{k}$) is called *committee* of size k, or simply k*-committee*. In what follows, the voters’ preferences are assumed to be additively separable. This reflects the idea that, when choosing between two committees, a voter prefers the one for which the sum of the voter’s scores^2^ of its candidates is higher.

Definition 2.4 Let $C,C^{\prime }\in A_{k}$. For i∈I  the binary relation ${⊵}_{i}$ on $A_{k}$ is defined by

$$C{⊵}_{i}C^{\prime }:\;⟺\;\underset{c\in C}{\sum }v_{i}\left(c\right)\geq \underset{c^{\prime }\in C^{\prime }}{\sum }v_{i}\left(c^{\prime }\right)\text{.}$$

If $C{▹}_{i}C^{\prime }$ we say voter i prefers C to $C^{\prime }$.

With abuse of notation, we abbreviate $v_{i}\left(C\right)≔\sum_{c\in C}v_{i}\left(c\right)$. Finally, a committee C is called weak Condorcet k-committee if there is no other k-committee that is preferred to C by a majority of voters; C is a strong Condorcet k-committee, if it beats each other committee in a pairwise majority comparison.

Definition 2.5 Let $C\in A_{k}$. C is a weak Condorcet k-committee, if $\left|\left\{i\in I:\;C{⊵}_{i}C^{\prime }\right\}\right|\geq \left|\left\{i\in I:\;C^{\prime }{⊵}_{i}C\right\}\right|$ for all $C^{\prime }\in A_{k}$. C is a strong Condorcet k-committee, if $\left|\left\{i\in I:\;C{⊵}_{i}C^{\prime }\right\}\right|\;>\;\left|\left\{i\in I:\;C^{\prime }{⊵}_{i}C\right\}\right|$ for all $C^{\prime }\in A_{k}$.

Clearly, a strong Condorcet committee is also a weak Condorcet committee. To simplify notation, for $C,C^{\prime }\in A_{k}$ we write $C►C^{\prime }$ if $\left|\left\{i\in I:\;C{⊵}_{i}C^{\prime }\right\}\right|>\left|\left\{i\in I:\;C^{\prime }{⊵}_{i}C\right\}\right|$.

Example 1 Given the voter preference profile π over the candidates {a,b,c,d,e,f,g} under 3-approval as displayed in Table 1, it is easy to check that both {a,b} and {a,c} are weak Condorcet 2-committees, whereas a strong Condorcet 2-committee does not exist.

However, {a,b,c} is not a weak Condorcet 3-committee — {a,d,g} is preferred to {a,b,c} by the voters 5–9, whereas {a,b,c} is preferred to {a,d,g} by the voters 1–4 only, and thus {a,d,g}►{a,b,c} holds. In particular, a weak (and hence a strong) Condorcet 3-committee does not exist in this example: We have {a,b,c}►C for all C∈{{a,b,d},{a,b,e},{a,b,f},{a,b,g},{a,c,d},{a,c,e},{a,c,f},{a,c,g},{a,d,e},{a,f,g}}; thus, none of these 10 sets can be a weak Condorcet 3-committee. In addition, we have $\left\{a,b,f\right\}►C^{\prime }$ for all $C^{\prime }\in \left\{\left\{a,d,f\right\},\left\{a,e,f\right\}\right\}$ and $\left\{a,b,g\right\}►C^{\prime \prime }$ for all $C^{\prime \prime }\in \left\{\left\{a,d,g\right\},\left\{a,e,g\right\}\right\}$; hence, none of these sets is a weak Condorcet 3-committee either. Therewith, there is no set containing a that forms a weak Condorcet 3-committee. Now, for any 3-element set S not containing a, one can find a 3-element set $S^{\prime }$ with $S^{\prime }►S$ by simply substituting an arbitrary element of S with a; hence, such a set S cannot be a weak Condorcet 3-committee either.

### 2.3 Problem formulation

We are now ready to state the weak Condorcet k-committee problem (CC) and the strong Condorcet k-committee problem (SCC), which is the task to decide if a given committee C is a weak (resp. strong) Condorcet k-committee.

Definition 2.6 **CC**

GIVEN: Set I of voters, set A of alternatives, voter preference profile π, voter $i^{\prime }s$ scoring function $v_{i}$ for all i∈I, k∈N, and k-committee $C\in A_{k}$.

QUESTION: Is C a weak Condorcet k-committee?

The problem SCC is defined by simply replacing in the above definition “weak Condorcet” by “strong Condorcet”.

Remark Note that CC and SCC belong to the class coNP. However, for k=1, CC (SCC) is equivalent to decide if a given candidate is a weak (strong) Condorcet winner. This question can be answered in polynomial time by executing the simple majority rule (for details concerning this voting rule see, e.g.,  Brams and Fishburn, 2002).

A natural problem related to these problems is the task of deciding whether there exists a weak respectively strong Condorcet k-committee.

Definition 2.7 **CC-Existence**

GIVEN: Set I of voters, set A of alternatives, voter preference profile π, voter $i^{\prime }s$ scoring function $v_{i}$ for all i∈I, and k∈N.

QUESTION: Is there a weak Condorcet k-committee?

Analogously, in the problem SCC-Existence we ask if there exists a strong Condorcet k-committee.

In the next section, we present a detailed computational complexity study of CC and SCC. In Section  4 we provide hardness results for CC-Existence and SCC-Existence.

## 3 Computational complexity of CC and SCC

In this section, we use the computational complexity status of the Min 2-Sat-3 problem, which is known to be NP-complete (and, in its optimization problem version, even APX-complete; see  Alimonti et al., 1997).

Definition 3.1 **Min 2-Sat-3**

GIVEN: Set X of variables, set C of (disjunctive) clauses over X such that every clause is made up of exactly two variables and the number of occurrences of each variable is bounded by 3, k∈N.

QUESTION: Is there a truth assignment τ for X that satisfies all clauses of C, such that the number of variables set to true under τ is at most k?

Note that in any instance of Min 2-Sat-3 as defined above the clauses are made up of variables and not of literals. I.e., there are no negated variables in an instance of Min 2-Sat-3.

In what follows, we identify a truth assignment τ with the set of variables set to true under τ. In particular, |τ| denotes the number of variables set to true under τ.

Remark 1 Let I be an instance of Min 2-Sat-3, and let n denote the number of variables and ℓ the number of clauses in I. A truth assignment that satisfies all clauses in I is called *satisfying truth assignment* for I. Throughout this paper we assume

(1) k<nandk<ℓ

since otherwise I is trivially satisfiable by a truth assignment τ with |τ|=k. In addition, we assume

(2) 3k≥ℓ

since otherwise I is not satisfiable by a truth assignment τ with |τ|≤k.

Remark 2 In this paper, we prove the coNP-completeness results for CC by showing that the complement of CC is NP-complete. In particular, we reduce Min 2-Sat-3 to the decision problem $\bar{\mathrm{CC}}$ whether, for a given committee C, there is a committee $C^{\prime }$ with $C^{\prime }►C$.

We begin the analysis of the complexity status of CC and SCC by considering Borda voting. Next, we state results for CC and SCC under approval voting, and conclude Section  3 with a discussion on CC and SCC in connection with multichotomous preferences.

### 3.1 CC and SCC under Borda voting

First, let us consider the case that the voters’ preference relations are strict orders on A, i.e., there are no ties in an individual’s ranking of the candidates. In such a case, it is an immediate approach to evaluate the value a committee has for a voter by means of scores used by positional scoring methods used in Social Choice Theory. The probably most famous representative of these methods is Borda voting; we get the following computational complexity result for CC under Borda voting.

Theorem 3.1 *The CC problem under Borda voting is* coNP*-complete, even if the preference profile consists of strict orders.*

Proof We prove the theorem by a polynomial transformation from an arbitrary instance I of Min 2-Sat-3. Let I be defined by a set C of ℓ clauses $C_{1},C_{2},\ldots ,C_{\ell }$ over the set $X≔\left\{x_{1},x_{2},\ldots ,x_{n}\right\}$ of variables, and let k∈N. W.l.o.g. we assume $C_{1}=\left(x_{1}\vee x_{3}\right)$. In general, for 1≤i≤ℓ we may write $C_{i}=\left(x_{i,1}\vee x_{i,2}\right)$ for some $x_{i,1},x_{i,2}\in X$.

From I we construct an instance U of $\bar{\mathrm{CC}}$ under Borda voting as follows:

• for every variable $x_{j}$ we introduce a candidate with the same label^3^
• we introduce $n^{2}$ candidates labelled $a_{1},a_{2},\ldots ,a_{n^{2}}$
• for every clause $C_{i}$ we introduce a voter $\gamma_{i}$ with the following ranking (see Table 2):
  – the two variables that make up $C_{i}$ are in the two top positions (for uniqueness of the profile they are ranked in alphabetical order)
  – the third to the $\left(n^{2}+2\right)$-nd position are taken by $a_{1},\ldots ,a_{n^{2}}$ in reversed alphabetical order
  – the last n−2 positions are, in alphabetical order, taken by the candidates $x_{j}$ not in $C_{i}$
• we introduce the voters $M_{1},\ldots ,M_{n^{2}-k}$ (see Table 2) such that
  – for each of these voters, the ranking restricted to the last n+k spots is
    $$x_{1}≻\cdots ≻x_{n}≻a_{1}≻\cdots ≻a_{k}$$
  – when restricted to the $n^{2}-k$ top positions, the preference profile made up of these voters builds a Condorcet-cycle among the candidates $\left\{a_{k+1},\ldots ,a_{n^{2}}\right\}$. I.e., for the $n^{2}-k$ top positions voter $M_{1}$ has $a_{k+1}≻a_{k+2}≻\cdots ≻a_{n^{2}}$, and the ranking of voter $M_{i+1},1\leq i\leq n^{2}-k-1$, on the first $n^{2}-k$ positions is derived from the one of $M_{i}$ by
    ∗ putting $M_{i}$’s top choice to the last position (i.e., position $n^{2}-k$) and
    ∗ for all $2\leq j\leq n^{2}-k$, lifting the candidate in position j to position j−1.
• we introduce the voters $\delta_{i},1\leq i\leq n^{2}-k+\ell -1$, whose ranking of the candidates (displayed in Table 3) is
  $$a_{1}≻a_{2}≻\cdots ≻a_{n^{2}}≻x_{1}≻x_{2}≻\cdots ≻x_{n}$$

That is, in instance U we are given the set of voters $I=\left\{\gamma_{1},\ldots ,\gamma_{\ell },M_{1},\ldots ,M_{n^{2}-k},\delta_{1},\ldots ,\delta_{n^{2}-k+\ell -1}\right\}$, the set of alternatives $A=\left\{x_{1},\ldots ,x_{n},a_{1},\ldots ,a_{n^{2}}\right\}$, and the voter preference profile π made up of the individuals’ preference relations defined above. Let

$$Z≔\left\{a_{1},\ldots ,a_{n^{2}}\right\}\text{.}$$

We now prove the following claim.

Claim 1 ∃ *a satisfying truth assignment* τ *for* I *with* |τ|≤k⟺ *In instance* U*there is a* $n^{2}$*-committee* D *such that* D►Z*, i.e., a majority of voters prefers* D *to* Z*.*

Proof of Claim 1 “⇒”: Let τ be a satisfying truth assignment with |τ|≤k. W.l.o.g. we can assume |τ|=k. Let $\left\{x_{\tau_{1}},\ldots ,x_{\tau_{k}}\right\}$ be the set of variables set to true under τ, i.e.,  $\tau =\left\{x_{\tau_{1}},\ldots ,x_{\tau_{k}}\right\}$. Let $D≔\left\{x_{\tau_{1}},\ldots ,x_{\tau_{k}}\right\}\cup \left\{a_{k+1},\ldots ,a_{n^{2}}\right\}$. Since τ is a satisfying truth assignment for I, for every voter $\gamma_{i},1\leq i\leq \ell$, at least one of her two top choices must be included in D. Therefore, for each voter $\gamma \in \left\{\gamma_{i}\;|1\leq i\leq \ell \right\}$ we have

$$v_{\gamma }\left(D\right)=\overset{k}{\underset{i=1}{\sum }}v_{\gamma }\left(x_{\tau_{i}}\right)+\overset{n^{2}}{\underset{i=k+1}{\sum }}v_{\gamma }\left(a_{i}\right)\geq \left[\left(n^{2}+n-2\right)+\overset{k-2}{\underset{i=0}{\sum }}i\right]+\overset{n^{2}}{\underset{i=k+1}{\sum }}v_{\gamma }\left(a_{i}\right)$$

whereas

$$v_{\gamma }\left(Z\right)=\overset{n^{2}}{\underset{i=1}{\sum }}v_{\gamma }\left(a_{i}\right)=\overset{n+k-3}{\underset{i=n-2}{\sum }}i+\overset{n^{2}}{\underset{i=k+1}{\sum }}v_{\gamma }\left(a_{i}\right)=\left(n-2\right)k+\overset{k-1}{\underset{i=1}{\sum }}i+\overset{n^{2}}{\underset{i=k+1}{\sum }}v_{\gamma }\left(a_{i}\right)$$

and hence

$$v_{\gamma }\left(D\right)-v_{\gamma }\left(Z\right)\geq \left(n^{2}+n-2\right)-\left(n-2\right)k-\left(k-1\right)=n^{2}-nk+n+k-1>0$$

where the last inequality follows from n>k (stated in  (1)). That is, all voters $\gamma \in \left\{\gamma_{i}\;|1\leq i\leq \ell \right\}$ prefer D to Z. Obviously, each of the voters $M_{1},\ldots ,M_{n^{2}-k}$ prefers D to Z as well, and hence D►Z holds.

“⇐”: On the other hand, let D►Z for some $D\in A_{n^{2}}$. Since each voter $\delta_{i}$ prefers Z to any other committee of size k, each of the remaining voters $\gamma_{1},\ldots ,\gamma_{\ell },M_{1},\ldots ,M_{n^{2}-k}$ must prefer D to Z.

Let $Q≔\left\{a_{k+1},\ldots ,a_{n^{2}}\right\}$. We now show that Q⊂D must hold. Assume the opposite, then there is an a∈Q∖D. By construction, there is a voter $M\in \left\{M_{1},\ldots ,M_{n^{2}-k}\right\}$ who ranks a on first position. Due to a∉D, we can upper bound $v_{M}\left(D\right)$ in terms of

$$v_{M}\left(D\right)\leq \underset{a_{j}\in Q\setminus \left\{a\right\}}{\sum }v_{M}\left(a_{j}\right)+\overset{k+1}{\underset{j=1}{\sum }}v_{M}\left(x_{j}\right)=\underset{a_{j}\in Q\setminus \left\{a\right\}}{\sum }v_{M}\left(a_{j}\right)+\overset{n+k-1}{\underset{j=n-1}{\sum }}j=\underset{a_{j}\in Q\setminus \left\{a\right\}}{\sum }v_{M}\left(a_{j}\right)+\left(n+k\right)\left(n+k-1\right)\frac{1}{2}-\left(n-2\right)\left(n-1\right)\frac{1}{2}=\underset{a_{j}\in Q\setminus \left\{a\right\}}{\sum }v_{M}\left(a_{j}\right)+\frac{1}{2}\left[\left(n^{2}+n\left(k-1\right)+kn+k\left(k-1\right)\right)-\left(n^{2}-3n+2\right)\right]=\underset{a_{j}\in Q\setminus \left\{a\right\}}{\sum }v_{M}\left(a_{j}\right)+kn+k\left(k-1\right)\frac{1}{2}+n-1$$

On the other hand we get

$$v_{M}\left(Z\right)=k\left(k-1\right)\frac{1}{2}+\underset{a_{j}\in Q\setminus \left\{a\right\}}{\sum }v_{M}\left(a_{j}\right)+\left(n^{2}+n-1\right)$$

which yields

$$v_{M}\left(Z\right)-v_{M}\left(D\right)=\left(n^{2}+n-1\right)-kn-n+1=n^{2}-kn>0$$

where again the last inequality follows from (1). This contradicts to the fact that voter M prefers D to Z.

Thus, Q⊂D must hold. However, this means |X∩D|≤k. Since every voter $\gamma_{1},\ldots ,\gamma_{\ell }$ prefers D to Z, for each of these voters at least one of her two top choices must be contained in D. These two facts together imply that I can be satisfied by setting at most k variables to true. ◊

Note that $\left|I\right|=2\left(n^{2}-k+\ell \right)-1$ and $\left|A\right|=n^{2}+n$, thus the transformation is polynomial because of k<n (stated in (1)). It is hence proven that an arbitrary instance I of Min 2-Sat-3 can be polynomially reduced to the complement of CC under Borda voting, which implies the coNP-completeness of CC under Borda voting. □

An analogous hardness result for SCC follows immediately.

Theorem 3.2 *The SCC problem under Borda voting is* coNP*-complete, even if the preference profile consists of strict orders.*

Proof Create instance $U^{\prime }$ of the complement $\bar{\mathrm{SCC}}$ of SCC from instance U of $\bar{\mathrm{CC}}$ under Borda voting (defined in the proof of Theorem 3.1) by adding voter $\delta_{n^{2}-k+\ell }$ whose ranking of the alternatives coincides with the one of the voters $\delta_{i},1\leq i\leq n^{2}-k+\ell -1$. Ceteris paribus, it is not hard to see that Z is a strong Condorcet k-committee in $U^{\prime }$ iff Z is a weak Condorcet committee in U. □

The negative results of Theorems 3.1 and 3.2 give rise to the question of the complexity status of CC and SCC in structurally simpler settings; i.e., when the information given is reduced and, instead of giving a full ranking of the candidates, the voters name only a set of approved candidates for example. Before turning our attention to such a setting (Section  3.2), we state the following lemma, which we will make use of in Section  4; it refers to instance U defined in the proof of Theorem 3.1.

Lemma 3.3 *In instance* U*, either* Z *is a weak Condorcet* $n^{2}$*-committee or a weak Condorcet* $n^{2}$*-committee does not exist.*

Proof It is sufficient to show that for any $n^{2}$-committee D≠Z, there is a $n^{2}$-committee $D^{\prime }►D$. If $Q=\left\{a_{k+1},\ldots ,a_{n^{2}}\right\}⊄D$, then, following the argumentation in the proof of Theorem 3.1, Z►D holds. Assume $Q=\left\{a_{k+1},\ldots ,a_{n^{2}}\right\}\subset D$. Since D≠Z and Q⊂Z, this means that for some 1≤i≤k, $a_{i}\in Z\setminus D$ holds. Now, for an arbitrary x∈D∖Z, we get $D\cup \left\{a_{i}\right\}\setminus \left\{x\right\}►D$, since the number of voters who prefer $a_{i}$ to x is at least $\left(\ell -3\right)+\left(n^{2}-k+\ell -1\right)$, whereas at most $3+\left(n^{2}-k\right)$ voters prefer x to $a_{i}$. □

### 3.2 CC and SCC under approval voting

In this section, we investigate the complexity of CC and SCC in the case that the voters are allowed to distinguish between “good” and “bad” candidates only (i.e., approval voting).

First, let each voter approve of exactly one candidate as it is the case in plurality voting.

Proposition 3.4 *CC under plurality voting can be solved in polynomial time. In addition, a weak Condorcet* k*-committee can be found in* O(|A|+r) *time.*

Proof We prove the second part of the proposition first. Rank the candidates a∈A in non-increasing order according to $\left|\left\{i:\;a\in S_{i}\right\}\right|$, and let G be a set consisting of the first k candidates in this ranking (ties are broken arbitrarily). Note that G is a solution of

(3) $$\arg\underset{Z\in A_{k}}{\max}\left|\left\{i\in I:\;S_{i}\subseteq Z\right\}\right|$$

Now assume that F►G for some $F\in A_{k}$. Then $\left|\left\{i\in I:\;S_{i}\subseteq F\right\}\right|>\left|\left\{i\in I:\;S_{i}\subseteq G\right\}\right|$, which contradicts to (3). That is, G is a weak Condorcet k-committee.

Let $A=\left\{a_{1},a_{2},\ldots ,a_{q}\right\}$ for some q∈N. The time needed to determine an array whose j-th entry contains $\left|\left\{i:\;a_{j}\in S_{i}\right\}\right|$ is in O(r), since in plurality voting we can assume that we do not need to scan the whole profile. By use of the selection algorithm (see  Fischetti and Martello, 1988), the time needed to determine G is in O(|A|). Thus, a weak Condorcet k-committee can be found in O(|A|+r) time.

For the first part of the proposition, let $C\in A_{k}$. It is sufficient to check if C is a solution of (3), i.e., if $\left|\left\{i\in I:\;S_{i}\subseteq C\right\}\right|=\left|\left\{i\in I:\;S_{i}\subseteq G\right\}\right|$. This, however, can be done in polynomial time. □

With analogous arguments, we get a positive, i.e., computational tractability result if each voter votes against exactly one candidate.

Proposition 3.5 *CC under antiplurality can be solved in polynomial time. In particular, a weak Condorcet* k*-committee can be found in* O(|A|+r) *time.*

Corollary 3.6 *Under plurality voting and antiplurality voting, SCC can be solved in polynomial time.*

Proof Follows straightforward from the proof of Proposition 3.4. In each case, it suffices to check if the given committee is the unique solution of (3) resp. of $\arg\min_{Z\in A_{k}}\left|\left\{i\in I:\;S_{i}^{c}\subseteq Z\right\}\right|$. □

Remark It should be noted that in the cases of plurality and antiplurality voting a weak Condorcet k-committee always exists, in contrast to strong Condorcet k-committees. However, in these cases, the existence of the latter can be checked easily.

Therewith, if each voter is allowed to choose only one “good” or one “bad” candidate, CC and SCC are easy to solve. However, this changes drastically if a voter is allowed to choose two “good” or two “bad” candidates, as the following theorems show.

Theorem 3.7 *Under*  2*-approval, both CC and SCC are* coNP*-complete.*

Proof We prove the theorem for CC first. Let I be an instance of Min 2-Sat-3 with ℓ clauses $C_{1},\ldots ,C_{\ell }$ over n variables $x_{1},\ldots ,x_{n}\in X$, let k∈N. Let $C_{i}=\left(x_{i,1}\vee x_{i,2}\right)$.

From I we construct the following instance V of $\bar{\mathrm{CC}}$ under 2-approval in two steps. First, we create the following set of alternatives, by

• introducing, for every variable $x_{j}$, a candidate with the same label
• introducing candidates $d_{1},d_{2},\ldots ,d_{n}$ and $f_{1},f_{2},\ldots ,f_{k}$

Thus, the set of candidates is $A≔\left\{x_{1},\ldots ,x_{n},d_{1},\ldots ,d_{n},f_{1},\ldots ,f_{k}\right\}$. Second, the voter preference profile π in instance V is created as follows (see also Table 4):

• for every clause $C_{i}$ we introduce k2 voters of type $\gamma_{i}$, such that a voter of type $\gamma_{i}$ approves of the two variables (candidates) that make up $C_{i}$
• for each i,1≤i≤n, we introduce 3kk2 voters of type $\delta_{i}$, such that a voter of type $\delta_{i}$ approves of $x_{i}$ and $d_{i}$
• we introduce $3k^{2}+\ell -1$ voters of type $F_{1,2}$, who approve of $f_{1}$ and $f_{2}$
• for every pair (i,j)≠(1,2) with i<j we introduce $3k^{2}+\ell$ voters of type $F_{i,j}$ who approve of $f_{i}$ and $f_{j}$

Let $F≔\left\{f_{1},\ldots ,f_{k}\right\}$.

Claim 2 ∃ *a satisfying truth assignment* τ *for* I *with* |τ|≤k⟺ *In instance* V*there is a committee* E *of size* k *such that* E►F*.*

Proof of Claim 2 “⇒”: Again, w.l.o.g. we assume |τ|=k. Let E≔τ, i.e.,  E consists of the variables set to true under τ. Then, the types of voters who prefer E to F are exactly k of the voter types $\delta_{j}$ and all the voters of type $\gamma_{i},1\leq i\leq \ell$. I.e., |{i:E▹iF}|=k⋅3kk2+ℓk2. On the other hand, the only voter types who prefer F to E are all the voter types $F_{i,j}$. Thus, |{i:F▹iE}|=(3k2+ℓ)k2−1. Hence, E►F.

“⇐”: Let E►F. Let s≔|E∩F|.

Assume s>0. Let p=k−s, i.e.,  p is the number of candidates in E∖F (and F∖E respectively). Note that s>0 and E≠F imply

(4) 1≤p≤k−1.

Then,

• at most p of the voter types $\delta_{i}$ and
• due to the fact that the number of occurrences of each variable is bounded by three, at most 3p of the voter types $\gamma_{i}$

prefer E to F. I.e.,

|{i:E▹iF}|≤p⋅3kk2+3p⋅k2=3pk2(k+1).

On the other hand, we need to count the number of voter types $F_{i,j}$ that prefer F to E. Since a voter of type $F_{i,j}$ prefers F to E iff $f_{i}\notin E$ or $f_{j}\notin E$ (or both), in a first step we are interested in the number ϵ of subsets of F of size 2 that contain at least one element of F∖E. Recall that p=|F∖E|. Thus, ϵ=p(k−p)+p2, where the first part is the number of subsets of size 2 that contain exactly one element of F∖E, and the second part is the number of subsets of F∖E of size 2. Now, because there are $3k^{2}+\ell$ voters of each type $F_{i,j}$, (i,j)≠(1,2), (and one voter less of type $F_{1,2}$) we get |{i:F▹iE}|≥(p(k−p)+p2)(3k2+ℓ)−1.

Now, E►F implies

3pk2(k+1)≥(p(k−p)+p2)(3k2+ℓ)

$$\Leftrightarrow \;3k\left(k-1\right)\left(k+1\right)\geq \left(2k-2p+p-1\right)\left(3k^{2}+\ell \right)$$

$$\Leftrightarrow \;3k\left(k-1\right)\left(k+1\right)\geq \left(2k-p-1\right)\left(3k^{2}+\ell \right)$$

$$\Rightarrow \;\;\;3k\left(k-1\right)\left(k+1\right)\geq k\left(3k^{2}+\ell \right)$$

$$\Leftrightarrow \;3k^{2}-3\geq 3k^{2}+\ell$$

⇔−3≥ℓ

where the fourth inequality follows from p≤k−1 (stated in (4)). However, −3≥ℓ contradicts to the definition of ℓ. Thus we must have s=0, i.e.,  E∩F=0̸.

Note that E∩F=0̸ means that |{i:F▹iE}|=(3k2+ℓ)k2−1. That is, E►F implies that the number of voters who prefer E to F must be at least (3k2+ℓ)k2.

Since $E\subset \left\{x_{i},d_{i}|1\leq i\leq n\right\}$ because of E∩F=0̸, each element of E is approved by exactly one of the voter types $\delta_{i}$. Hence, |E|=k implies that at most k of the voter types $\delta_{i}$ prefer E to F. Thus, all the voter types $\gamma_{i}$ must prefer E to F, since otherwise |{i:E▹iF}|≤(3k2+ℓ−1)k2. Therefore, for each clause $C_{i}$ at least one of the variables $x_{i,1},x_{i,2}$ must be contained in E. Hence, $\tau ≔E\setminus \left\{d_{1},\ldots ,d_{n}\right\}$ is a satisfying truth assignment for I. Due to |E|=k we finally get |τ|≤k. ◊

Note that in instance V the number of candidates is 2n+k and the number of voters is 3kk2n+k2ℓ+[(3k2+ℓ)k2−1]=(3kn+3k2+2ℓ)k2−1. Since k2=k(k−1)2, the number of candidates and voters is polynomial in n,k and ℓ. Therefore, the above reduction from Min 2-Sat-3 is polynomial due to (1) and the theorem is proven for CC.

For SCC, consider instance $V^{\prime }$ derived from V by adding one voter of type $F_{1,2}$. Analogously to the above proof it follows that F is a strong Condorcet k-committee in $V^{\prime }$ iff I is a “no”-instance of Min 2-Sat-3. □

We can derive a lemma to be used in Section  4 which refers to instance V defined in the above proof.

Lemma 3.8 *In instance* V*, either* F *is a weak Condorcet* k*-committee or a weak Condorcet* k*-committee does not exist.*

Proof For D≠F, we show that D cannot be a weak Condorcet k-committee. Let x∈D∖F and f∈F∖D. Recall that x is approved of by at most 3kk2+3k2 voters, whereas f is approved of by at least $\left(3k^{2}+\ell \right)\cdot \left(k-1\right)-1$ voters. Note that

(3k2+ℓ)⋅(k−1)−1>3kk2+3k2

$$\Leftrightarrow \;\left(3k^{2}+\ell \right)\cdot \left(k-1\right)-1>3\left(k+1\right)\frac{k\left(k-1\right)}{2}$$

$$\Leftrightarrow \;3k^{3}-3k^{2}+\ell \left(k-1\right)-1>\frac{3}{2}k\left(k^{2}-1\right)$$

$$\Leftrightarrow \;3k^{3}-3k^{2}+\ell \left(k-1\right)-1>\frac{3}{2}k^{3}-\frac{3}{2}k$$

$$\Leftrightarrow \;\frac{3}{2}k^{3}-3k^{2}+\frac{3}{2}k+\ell \left(k-1\right)-1>0$$

holds for any k>1, which means that D∪{f}∖{x}►D is satisfied. □

In addition, from Theorem 3.7 we can immediately derive two corollaries. E.g., it follows that CC and SCC remain coNP-complete, even if restricted to voters’ scoring functions that map into {0,1}; this implies the following corollary.

Corollary 3.9 *Both CC and SCC are* coNP*-complete in the strong sense.*

As a consequence of Theorem 3.7 we get an analogous complexity result for CC (resp. SCC) under t-approval for any t≥2.

Corollary 3.10 *Both CC and SCC are* coNP*-complete under* t*-approval for any fixed* t≥2,t∈N*.*

Proof We prove the corollary for CC (for SCC it follows analogously). The case t=2 is covered by Theorem 3.7.

For t≥3, the proof follows from the one of Theorem 3.7 by a slight modification of the instance V. In particular, we introduce t−2 additional candidates $y_{i},1\leq i\leq t-2$, and add them to each voter’s set of approved edges. Call the new candidate set $A^{\prime }≔A\cup \left\{y_{1},\ldots ,y_{t-2}\right\}$ and the new instance Z. Let $F^{\prime }≔\left\{f_{1},\ldots ,f_{k},y_{1},\ldots ,y_{t-2}\right\}$ and let $Y≔\left\{y_{1},\ldots ,y_{t-2}\right\}$. Note that for each voter $i,Z{⊵}_{i}F^{\prime }\Leftrightarrow \left|Z\cap S_{i}\right|\geq \left|F^{\prime }\cap S_{i}\right|$ holds for any $Z\in A_{k+t-2}^{\prime }$ (where $S_{i}$ relates to instance Z).

We show that F is a weak Condorcet k-committee in V iff $F^{\prime }$ is a weak Condorcet (k+t−2)-committee in Z by proving the following claim.

Claim 3 ∃E *such that* E►F *in* $V\Leftrightarrow \exists \;E^{\prime }$ *such that* $E^{\prime }►F^{\prime }$ *in* Z*.*

Proof of Claim 3 “⇒”: Given such a committee $E,E^{\prime }≔E\cup Y$ obviously satisfies $E^{\prime }►F^{\prime }$.

“⇐”: Let $E^{\prime }►F^{\prime }$ for some $E^{\prime }\in A_{k+t-2}^{\prime }$. If $Y\subset E^{\prime }$, then $\left(E^{\prime }\setminus Y\right)►F$ in instance V because of $Y\subset F^{\prime }$. Assume that there is exactly one element y∈Y that is not contained in $E^{\prime }$. For some $h\in E^{\prime }\setminus Y$, let $E^{\prime \prime }=E^{\prime }\cup \left\{y\right\}\setminus \left\{h\right\}$. Now, since each voter approves of y, we have $\left|E^{\prime \prime }\cap S_{i}\right|>\left|E^{\prime }\cap S_{i}\right|$ if $h\notin S_{i}$ and $\left|E^{\prime \prime }\cap S_{i}\right|=\left|E^{\prime }\cap S_{i}\right|$ if $h\in S_{i}$. Thus, if i prefers $E^{\prime }$ to $F^{\prime }$, she prefers $E^{\prime \prime }$ to $F^{\prime }$ as well; if she is indifferent between $E^{\prime }$ and $F^{\prime },E^{\prime \prime }{⊵}_{i}F^{\prime }$ holds.

I.e., $E^{\prime }►F^{\prime }$ implies the existence of a committee $E^{\prime \prime }$ such that $Y\subset E^{\prime \prime }$ and $E^{\prime \prime }►F^{\prime }$. As above, this means that $\left(E^{\prime \prime }\setminus Y\right)►F$ must hold in instance V.

Finally, there cannot exist two or more elements in Y that are not contained in $E^{\prime }$, since otherwise no voter would prefer $E^{\prime }$ to $F^{\prime }$ and thus $E^{\prime }►F^{\prime }$ would not hold. □

Turning our attention to CC and SCC under 2-veto, it turns out that the problems show the same computational complexity behaviour as in the case of 2-approval.

Theorem 3.11 *Under*  2*-veto, both CC and SCC are* coNP*-complete.*

Proof We give a proof for CC first. Let I be an instance of Min 2-Sat-3 with a set C of ℓ clauses $C_{1},\ldots ,C_{\ell }$ over a set $X=\left\{x_{1},\ldots ,x_{n}\right\}$ of variables, and let k∈N. We restrict the attention to instances with k≥9 (Min 2-Sat-3 remains NP-complete under this restriction). Let

w≔k(n−k)+k2−ℓ.

Now in order to construct from I the instance W of $\bar{\mathrm{CC}}$ under 2-veto we introduce

• for every variable $x_{j}$ a candidate with the same label
• candidates $d,f_{1},\ldots ,f_{k}$
• for each 1≤i≤k,w+1 voters disapproving of candidates $f_{i}$ and d (“type $F_{i}$ voters”)
• for each (i,j),i<j, such that $\left(x_{i}\vee x_{j}\right)\notin C$, k voters disapproving of candidates $x_{i}$ and $x_{j}$ (“type $\chi_{i,j}$ voters”)

Note that we have n2−ℓ different types $\chi_{i,j}$, one for each set $\left\{x_{i},x_{j}\right\}$ that does not make up one of the clauses $C_{1},\ldots ,C_{\ell }$. Let $F≔\left\{f_{1},\ldots ,f_{k}\right\}$.

Claim 4 ∃ *a satisfying truth assignment* τ *for* I *with* |τ|≤k⟺ *In instance* W*there is a committee* E *of size* k *such that* E►F*.*

Proof of Claim 4 “⇒”: Again, w.l.o.g. we assume |τ|=k. Let E≔τ. It is straightforward to see that the set of voters who prefer E to F consists of all voters of type $F_{i},1\leq i\leq k$. Hence

(5) $$\left|\left\{i:\;E{▹}_{i}F\right\}\right|=\left(w+1\right)k\text{.}$$

On the other hand, the set of voters who prefer F to E consists of all the voters of the types $\chi_{i,j}$ who disapprove of at least one variable (alternative) $x_{j}$ set to true under τ. Since |E|=k, the number of subsets of X of size 2 that contain at least one variable of E is exactly k(n−k)+k2. Since τ is a satisfying truth assignment, each of the ℓ clauses contains at least one variable set to true; in other words, each clause is made up by a set {e,g} where at least one of the candidates e and g is contained in E. By construction, this means that the number of voter types who disapprove of at least one variable contained in E is k(n−k)+k2−ℓ. Thus,

(6) |{i:F▹iE}|=(k(n−k)+k2−ℓ)k=wk.

Taking (5) and (6) together yields E►F.

“⇐”: Let E►F. Clearly, d∉E, since otherwise no voter would prefer E to F. Let s≔|E∩F|.

Again, let

p≔k−s.

I.e., p is the number of candidates in E∖F (and F∖E resp.). Since d∉E,p also corresponds to the number of candidates in E∩X. Clearly, E≠F implies

(7) p≥1.

Obviously, we get

(8) $$\left|\left\{i:\;E{▹}_{i}F\right\}\right|=\left(w+1\right)p$$

because of p=|F∖E|. Let τ≔E∩X. Note that

(9) |τ|=p=k−s

*Case* 1: s≥4. Recall that the number of occurrences of a variable in I is bounded by three. Therefore, in I at most 3p clauses can be satisfied by the truth assignment τ. That is, at least (p(n−p)+p2−3p) voter types prefer F to E, i.e., 

|{i:F▹iE}|≥(p(n−p)+p2−3p)k.

With (8), E►F thus implies

(w+1)p>(p(n−p)+p2−3p)k

$$\Leftrightarrow \;w+1>\left(n-p+\frac{p-1}{2}-3\right)k$$

$$\Leftrightarrow \;k\left[\left(n-k+\frac{k-1}{2}\right)-\left(n-p+\frac{p-1}{2}-3\right)\right]>\ell -1$$

$$\Leftrightarrow \;k\left(p-k+\frac{k-p}{2}+3\right)>\ell -1$$

$$\Leftrightarrow \;k\left(\frac{p-k}{2}+3\right)>\ell -1$$

where the second inequality trivially follows from p≥1 (stated in (7)). Since p≤k−4 due to s≥4, the last inequality implies k>ℓ−1, in contradiction to k<ℓ. Thus, we must have s≤3 (Case 2 below).

*Case* 2: s≤3. We now show that in instance I, τ must satisfy all clauses. Assume the opposite holds, i.e., assume τ to satisfy at most ℓ−1 clauses. Then we get

(10) |{i:F▹iE}|≥(p(n−p)+p2−(ℓ−1))k.

With (8) and (10), E►F yields

(11) (w+1)p>(p(n−p)+p2−(ℓ−1))k

⇔p(k(n−k)+k2−ℓ+1)>(p(n−p)+p2−(ℓ−1))k

$$\Leftrightarrow \;p\left(kn-k^{2}+\frac{k^{2}-k}{2}-\ell +1\right)>\left(pn-p^{2}+\frac{p^{2}-p}{2}-\ell +1\right)k$$

$$\Leftrightarrow \;pkn-p\frac{k^{2}}{2}-p\frac{k}{2}-p\ell +p>pkn-k\frac{p^{2}}{2}-k\frac{p}{2}-k\ell +k$$

$$\Leftrightarrow \;\ell \left(k-p\right)>p\frac{k^{2}}{2}-k\frac{p^{2}}{2}+k-p$$

$$\Leftrightarrow \;\ell \left(k-p\right)>\frac{pk}{2}\left(k-p\right)+k-p\text{.}$$

Recall that s=k−p. Since the last inequality contradicts to s=0, s∈{1,2,3} must hold. Dividing the last inequality in (11) by (k−p) yields

(12) $$\ell >\frac{pk}{2}+1\text{.}$$

However, we have $\frac{pk}{2}+1=\frac{k-s}{2}k+1=\frac{1}{2}\left(k^{2}-sk\right)+1$. For k≥9 and s∈{1,2,3}

$$\frac{1}{2}\left(k^{2}-sk\right)+1>\frac{1}{2}\left(k^{2}-sk\right)\geq 3k$$

holds, and thus

$$\frac{pk}{2}+1>3k$$

is satisfied. With (12), this means ℓ>3k holds, in contradiction to (2). Thus, in instance I, τ must satisfy all ℓ clauses. ◊

Because in instance W there are n+k+1 candidates and k(n2−ℓ)+k(k(n−k)+k2−ℓ+1)∈O(kn2) voters, the above reduction from Min 2-Sat-3 is polynomial, which completes the proof.

For SCC, construct instance $W^{\prime }$ from W by removing one voter of type $F_{i}$, for each 1≤i≤k. It is not hard to verify that I is a “no”-instance of Min 2-Sat-3 iff F is a strong Condorcet k-committee. □

Lemma 3.12 *In instance* W*, either* F *is a weak Condorcet committee or such a committee does not exist.*

Proof Let G≠F. We show that G cannot be a weak Condorcet committee. Let f∈F∖G. If d∈G∖F, then obviously G∪{f}∖{d}►G holds. Assume d∉G∖F, then for some x∈X,x∈G∖F holds. Recall that x is disapproved of by at least k((n−1)−3)=k(n−4) voters, whereas f is disapproved by exactly w+1=k(n−k)+k2−ℓ+1 voters. Clearly,

$$k\left(n-4\right)>k\left(n-k+\frac{k-1}{2}\right)-\ell +1$$

$$\Leftrightarrow \;kn-4k>kn+k\left(\frac{-k-1}{2}\right)-\ell +1$$

$$\Leftrightarrow \;0>k\left(\frac{-k+7}{2}\right)-\ell +1$$

where the last inequality holds for any k>5 because of ℓ>k. Thus, G∪{f}∖{x}►G holds. □

Corollary 3.13 *Both CC and SCC are* coNP*-complete under* t*-veto for any fixed* t≥2,t∈N*.*

Proof Ceteris paribus, the proof follows from the one of Theorem 3.11 by introducing t−2 additional candidates $y_{i},1\leq i\leq t-2$, and inserting them in each voter’s set of disapproved edges. □

### 3.3 CC and SCC with multichotomous preferences

In the two previous sections, computational complexity results for CC and SCC under Borda voting and approval voting have been presented. In particular, the cases that individual preferences are expressed by linear orders and dichotomous preferences have been considered, and a complete analysis in terms of complexity results of the latter case has been established.

However, using Theorem 3.7, it can be shown that CC and SCC remain coNP-complete in case of multichotomous preferences, even if the number of preference classes q is the same for every voter (Corollary 3.14).

Within this section, for i∈I and 1≤j,h≤q, let $A_{j}^{i}$ be the j-th preference class of voter i such that $a\in A_{j}^{i}$ and $b\in A_{h}^{i}$ with h>j is equivalent to $a{≻}_{i}b$. The classes $A_{1}^{i}$ and $A_{q}^{i}$ are called voter i’s *top class* and *bottom class* respectively.

Corollary 3.14 *Both CC and SCC are* coNP*-complete if the voters have multichotomous preferences, even if each* ${≿}_{i}$*,* 1≤i≤r*, induces the same number* q *of preference classes for some* q≥2*.*

Proof We prove the corollary for CC; the hardness of SCC follows analogously. The case q=2 (dichotomous preferences) is covered by Theorem 3.7.

Let q≥3. Then the coNP-completeness result can be derived from the proof of Theorem 3.7 as follows. In order to derive the profile $\pi^{\prime }$ from the voter profile π we

• introduce q−2 additional candidates $t_{1},\ldots ,t_{q-2}$
• let, for i∈I,
  $$A_{j}^{i}=\left\{ \begin{array}{cc} t_{j} & \text{if }1\leq j\leq q-2\\ S_{i} & \text{if }j=q-1\\ S_{i}^{c} & \text{if }j=q \end{array}\right.$$

Let the instance $V^{\prime }$ of $\bar{\mathrm{CC}}$ consist of the set $I^{\prime }≔I$ of voters, the set $A^{\prime }≔A\cup \left\{t_{j}|1\leq j\leq q-2\right\}$ of alternatives and the voter preference profile $\pi^{\prime }$. In addition, consider the following voting rule R using a scoring function that assigns, for each voter i,q−j points to a candidate in $A_{j}^{i}$, i.e., let $v_{i}\left(a\right)=q-j\text{ if }a\in A_{j}^{i}$, for $i\in I^{\prime }$ and 1≤j≤q. Let $F^{\prime }≔\left\{f_{1},\ldots ,f_{k},t_{1},\ldots ,t_{q-2}\right\}$.

Claim 5 *The following two decision problems are equivalent:*

(P1) *GIVEN: Instance* V *of* $\bar{\mathrm{CC}}$ *under*  2*-approval (as in the proof of*Theorem  3.7*)*
*QUESTION: Is there a committee* E *of size* k *such that* E►F*, where* $F=\left\{f_{1},\ldots ,f_{k}\right\}$*?*
(P2) *GIVEN: Instance* $V^{\prime }$ *of* $\bar{\mathrm{CC}}$ *under rule* R
*QUESTION: Is there a committee* $E^{\prime }$ *of size* k+q−2 *such that* $E^{\prime }►F^{\prime }$*?*

Proof of Claim 5 Let $E^{\prime }$ be a committee of size k+q−2 such that $E^{\prime }►F^{\prime }$. Clearly, $t_{j}\in E^{\prime }$ must hold for all 1≤j≤q−2, since otherwise no voter prefers $E^{\prime }$ to $F^{\prime }$. Thus, the claim follows. ◊

From the above claim we can conclude the theorem by the proof of Theorem 3.7. □

For trichotomous preferences, we may consider the following voting rule.

Definition 3.2 Let ${≿}_{i}$ be trichotomous for all i∈I. Under rule r, voter i’s scoring function is

$$r_{i}\left(j\right)=\left\{ \begin{array}{cc} 2 & \text{for }j\in A_{1}^{i}\\ 1 & \text{for }j\in A_{2}^{i}\\ 0 & \text{for }j\in A_{3}^{i}\text{.} \end{array}\right.$$

In case of trichotomous preferences, from (the proof of) Corollary 3.14 it follows that CC and SCC are coNP-complete for r, even if the number of candidates in the top class is restricted to be one. An interesting case arises when the number of elements in the top class and the number of elements in the last class is one.^4^ For this case, the computational complexity of CC and SCC under rule r is open.

## 4 Hardness of CC-Existence and SCC-Existence

In this section, we consider the problems of deciding whether or not a weak respectively strong Condorcet k-committee exists. Clearly, both CC-Existence and SCC-Existence belong to the class $\Sigma_{2}^{p}$. As a consequence of Lemmata 3.3, 3.8 and 3.12, the coNP-hardness results of CC imply that CC-Existence is coNP-hard, even under Borda voting, t-approval or t-veto for t≥2 (stated in Theorem 4.3). It is not hard to see that analogues of the above lemmata can be formulated for strong Condorcet k-committees (and the respective instances). Thus, also SCC-Existence is coNP-hard under Borda voting, t-approval or t-veto (t≥2).

Theorem 4.1 *SCC-Existence is* coNP*-hard, even under Borda voting or approval voting.*

In particular, for approval voting SCC-Existence is coNP-complete.

Corollary 4.2 *For approval voting, SCC-Existence is* coNP*-complete.*

Proof Due to Theorem 4.1 it is sufficient to show that in the case of approval voting SCC-Existence is in coNP. Given an instance I of SCC-Existence with set A of alternatives, set I of voters and k∈N, determine for each a∈A the value $s\left(a\right)≔\left|\left\{i:\;a\in S_{i}\right\}\right|$. Label the candidates with $a_{j},1\leq j\leq \left|A\right|$, in a way such that $s\left(a_{j}\right)\geq s\left(a_{j+1}\right)$ for all 1≤j≤|A|−1 holds (this labelling is not necessarily unique). Let $G≔\left\{a_{1},a_{2},\ldots ,a_{k}\right\}$. Clearly, by a simple exchange argument it follows that a committee C≠G cannot be a strong Condorcet k-committee. Hence, for approval voting SCC-Existence reduces to the problem of deciding if G is a strong Condorcet k-committee. Thus, for approval voting SCC-Existence is in coNP. □

Theorem 4.3 *CC-Existence is* coNP*-hard, even under Borda voting or approval voting.*

In fact, it turns out that CC-Existence is both coNP-hard and NP-hard. In particular, assuming NP≠coNP, in the case of approval voting CC-Existence and SCC-Existence show a different computational complexity behaviour.

Theorem 4.4 *CC-Existence is* NP*-hard, even under*  2*-approval voting.*

Proof We provide a reduction from Independent Set (IS). The IS problem is the following decision problem: Given an undirected graph G=(V,E) and a number k, does G admit an independent set of size at least k? It is known that IS is NP-complete even for cubic graphs (Garey and Johnson, 1977).

For an instance 〈G,k〉 of IS where G is a cubic graph, we can assume that 3k≤|E| holds, since obviously otherwise 〈G,k〉 is a “no”-instance. Given such an instance S=〈G,k〉 with |E|>12, we construct an instance C of CC-Existence as follows: Set A≔V and I≔E, and let π be such that, for each e={g,h}∈E, voter e approves of the candidates g,h exclusively. Note that since G is a cubic graph, each candidate is approved of by exactly three voters. Let m≔|E|. We show that S is a “yes”-instance of IS iff C admits a weak Condorcet k-committee.

If S is a “yes”-instance of IS, then there is a subset $V^{\prime }\subseteq V$ of size k such that no two vertices in $V^{\prime }$ are joined by an edge in E; i.e.,  $A^{\prime }≔V^{\prime }$ is a k-committee such that no two candidates are approved of by the same voter. In other words, there are exactly 3k voters i∈I with $v_{i}\left(A^{\prime }\right)=1$, whereas for the remaining m−3k voters i we have $v_{i}\left(A^{\prime }\right)=0$. We argue that $A^{\prime }$ is a weak Condorcet k-committee. Let $B\in A_{k}$. Obviously, if B constitutes an independent set of G, then $B∼A^{\prime }$ holds. Assume that B is not an independent set of G. Let $I_{r}$ denote the set of voters i∈I for which $v_{i}\left(B\right)=r,r\in \left\{0,1,2\right\}$. Clearly,

(13) $$2\left|I_{2}\right|+\left|I_{1}\right|=3k$$

because G is cubic. Now, $\left|\left\{i\in I\setminus I_{2}:A^{\prime }{▹}_{i}B\right\}\right|-\left|\left\{i\in I\setminus I_{2}:B{▹}_{i}A^{\prime }\right\}\right|\geq 3k-\left|I_{2}\right|-\left|I_{1}\right|$. On the other hand, $\left|\left\{i\in I_{2}:A^{\prime }{▹}_{i}B\right\}\right|-\left|\left\{i\in I_{2}:B{▹}_{i}A^{\prime }\right\}\right|=0-\left|I_{2}\right|=-\left|I_{2}\right|$. In total, with (13) we get $\left|\left\{i\in I:A^{\prime }{▹}_{i}B\right\}\right|-\left|\left\{i\in I:B{▹}_{i}A^{\prime }\right\}\right|\geq 3k-\left|I_{2}\right|-\left|I_{1}\right|-\left|I_{2}\right|=0$. I.e., $A^{\prime }$ is a weak Condorcet k-committee.

If S is a “no”-instance of IS, then for each $B\in A_{k}$ there is at least one voter i∈I for which $v_{i}\left(B\right)=2$, i.e.,  $S_{i}=\left\{b_{1},b_{2}\right\}$ for some $b_{1},b_{2}\in B$. From the degree sum formula we know that 2m=3|V|, or equivalently, $\left|V\right|=\frac{2}{3}m$ holds. Now, 3k≤m implies that $\left|A\setminus B\right|=\left|V\right|-k\geq \frac{2}{3}m-\frac{1}{3}m=\frac{1}{3}m$. With m>12, this yields |A∖B|≥5. Since deg(v)=3 for each v∈V, this means that there must be two vertices $g_{1},g_{2}\in A\setminus B$ that are not adjacent in G. Let $C≔B\cup \left\{g_{1},g_{2}\right\}\setminus \left\{b_{1},b_{2}\right\}$, and let $q≔\left|\left\{i\in I:\left\{g_{1},g_{2}\right\}{∼}_{i}\left\{b_{1},b_{2}\right\}\right\}\right|$. Then, $\left|\left\{i\in I:C{▹}_{i}B\right\}\right|-\left|\left\{i\in I:B{▹}_{i}C\right\}\right|=\left(6-q\right)-\left(5-q\right)=1$. Hence, for each $B\in A_{k}$ there is a $C\in A_{k}$ with C►B. Thus, a weak Condorcet k-committee does not exist. □

Our results have shown that CC-Existence is both NP-hard and coNP-hard, and SCC-Existence is coNP-hard. While in the special case of approval voting the latter problem is coNP-complete, we suspect that in general each problem is at least DP-hard–and, in particular, believe that this is the case for CC-Existence under approval voting already–but we do not have a proof.

## 5 Conclusion

With respect to classic voting schemes adapted from Social Choice Theory, we have analysed the computational complexity involved in deciding if a given committee is at least as good as (resp. better than) every other committee in a pairwise majority comparison. In particular, we have established the sharp separation line between computationally tractable and intractable instances. As our results show, if the voters vote for or against exactly one candidate it can be determined efficiently if a given committee is a weak or strong Condorcet k-committee; more than this, it is even possible in polynomial time (i) to find a weak Condorcet k-committee, and (ii) to decide if a strong Condorcet k-committee exists, and to find such a committee if it exists. On the other hand, CC and SCC are computationally hard to solve not only for Borda voting — as soon as the voters distinguish more than one alternative in a positive or negative sense, the problems become coNP-complete. Therewith, we have established the complexity status of CC and SCC for the most reasonable voting rules.

As it turns out, also the problem SCC-Existence is coNP-hard. In addition, CC-Existence is both NP-hard and coNP-hard. While these results establish lower bounds on the complexity of these problems, a finer placement is still open.

## Figures and Tables

**Table 1:** Voter preference profile π for 9 voters under 3-approval.

voters	1, 2, 3, 4	5, 6, 7	8, 9	scores
candidates	a	a	a	**1**
	b	d	f	**1**
	c	e	g	**1**
	d	b	b	**0**
	e	c	c	⋮
	f	f	d	
	g	g	e	**0**

**Table 2:** Voter preference profile π for voters $\gamma_{i},1\leq i\leq \ell$, and $M_{j},1\leq j\leq n^{2}-k$, in instance U.

$\gamma_{1}$	$\gamma_{2}$	…	$\gamma_{\ell }$	Borda count
$x_{1}$	$x_{2,1}$		$x_{\ell ,1}$	$n^{2}+n-1$
$x_{3}$	$x_{2,2}$		$x_{\ell ,2}$	$n^{2}+n-2$
$a_{n^{2}}$	$a_{n^{2}}$	$a_{n^{2}}$	$a_{n^{2}}$	$n^{2}+n-3$
$a_{n^{2}-1}$	$a_{n^{2}-1}$	$a_{n^{2}-1}$	$a_{n^{2}-1}$	⋮
⋮	⋮	⋮	⋮	
$a_{1}$	$a_{1}$	$a_{1}$	$a_{1}$	n−2
$x_{2}$	⋮	⋮	⋮	n−3
$x_{4}$				⋮
$x_{5}$				
⋮				
$x_{n}$				0


$M_{1}$	$M_{2}$	…	$M_{n^{2}-k}$	Borda count
$a_{k+1}$	$a_{k+2}$		$a_{n^{2}}$	$n^{2}+n-1$
$a_{k+2}$	$a_{k+3}$		$a_{k+1}$	$n^{2}+n-2$
⋮	⋮		⋮	⋮
$a_{n^{2}-1}$	$a_{n^{2}}$		$a_{n^{2}-2}$	n+k+1
$a_{n^{2}}$	$a_{k+1}$		$a_{n^{2}-1}$	n+k
$x_{1}$	$x_{1}$		$x_{1}$	n+k−1
$x_{2}$	$x_{2}$		$x_{2}$	n+k−2
⋮	⋮		⋮	⋮
$x_{n}$	$x_{n}$		$x_{n}$	k
$a_{1}$	$a_{1}$		$a_{1}$	k−1
⋮	⋮		⋮	⋮
$a_{k}$	$a_{k}$		$a_{k}$	0

**Table 3:** Voter preference profile π for the voters $\delta_{i}$ in instance U.

$\delta_{i}$	Borda count
$a_{1}$	$n^{2}+n-1$
$a_{2}$	$n^{2}+n-2$
⋮	⋮
$a_{n^{2}}$	n
$x_{1}$	n−1
$x_{2}$	n−2
⋮	⋮
$x_{n-1}$	1
$x_{n}$	0

**Table 4:** Voter types and corresponding approval counts in instance V.

$\delta_{i}$	
candidate	appr. count
$x_{i}$	**1**
$d_{i}$	**1**

r	**0**
e	⋮
s	
t	**0**


$\gamma_{i}$	
candidate	appr. count
$x_{i,1}$	**1**
$x_{i,2}$	**1**

r	**0**
e	⋮
s	
t	**0**


$F_{i,j}$	
candidate	appr. count
$f_{i}$	**1**
$f_{j}$	**1**

r	**0**
e	⋮
s	
t	**0**

## Footnotes

1 In general, Borda voting is not restricted to, but typically defined for the case that the individual rankings are strict orders over the set of alternatives. In Definition 2.3, Borda voting can easily be extended to weak orders, e.g., by replacing ${≻}_{i}$ with ${≿}_{i}$.

2 i.e., the values of the voter’s scoring function.

3 Within the proofs of this paper, we identify a variable with the candidate having the same label. Therefore, we will use the terms “variable $x_{j}$” and “candidate $x_{j}$” interchangeably.

4 In that case, r corresponds to the pure scoring rule with vector (2,1,…,1,0) as considered, e.g., by Betzler and Dorn (2010) and Baumeister and Rothe (2012) in the context of the Possible Winner Problem.
